# Therapeutic effects of radix dipsaci, pyrola herb, and cynomorium songaricum on bone metabolism of ovariectomized rats

**DOI:** 10.1186/1472-6882-12-67

**Published:** 2012-05-28

**Authors:** Meijie Liu, Gary Guishan Xiao, Peijing Rong, Zhiguo Zhang, Jiazi Dong, Hongyan Zhao, Honghong Li, Yan Li, Jinghua Pan, Hong Liu, Wenlai Wang, Qinglin Zha, Dahong Ju

**Affiliations:** 1 Institute of Basic Theory, China Academy of Chinese Medical Sciences, South Small Street 16, Dongzhimennei, Dongcheng District, Beijing 100700, Peoples Republic of ChineChina; 2 Functional Genomics & Proteomics Laboratory, Osteoporosis Research Center, Creighton University Medical Center, 601N 30th Street, Suite 6730, Omaha, NE 68131, USA; 3 Institute of Acupuncture Moxibustion, China Academy of Chinese Medical Sciences, South Small Street 16, Dongzhimennei, Dongcheng District, Beijing 100700, China; 4 Hospital of T.C.M, Shijingshan District, Bajiao North Road, Shijingshan District, Beijing 100041, China; 5 Jiangxi College of Traditional Chinese Medicine, Yunwan Road 18, Wanli District, Nanchang City, Jiangxi 330004, China

## Abstract

**Background:**

The objective of this study was to evaluate the effects of herbal medicines, such as *Radix Dipsaci* (RDD), *Pyrola Herb* (PHD), and *Cynomorium songaricum* decoction (CSD), on osteoporotic rats induced by ovariectomy (OVX).

**Methods:**

OVX or sham operations were performed on 69 virgin Wistar rats that were divided into six groups: sham (sham, n = 12), OVX control group (OVX, n = 12), and OVX rats with treatments (diethylstilbestrol, E2, n = 12; RDD, n = 11, PHD, n = 11, and CSD, n = 11). Non-surgical rats served as normal control (NC, n = 12). The treatments began four weeks after surgery and lasted for 12 weeks. Bone mass and bone turnover were analyzed by histomorphometry. Levels of protein expression and mRNA of OPG and RANKL in osteoblasts (OB) and bone marrow stromal cells (bMSC) were evaluated by immunohistochemistry and *in situ* hybridization.

**Results:**

Compared to NC and sham rats, trabecular bone formation was significantly reduced in OVX rats, but restored in E2-treated rats. Treatment with either RDD or PHD enhanced trabecular bone formation remarkably. No significant change of bone formation was observed in CSD-treated rats. OPG expression of protein and mRNA was reduced significantly in OB and bMSC of OVX control rats. RANKL expression of protein and mRNA was increased significantly in OB and bMSC of OVX control rats. These effects were substantially reversed (increased in OPG and decreased in RANKL) by treatment with E2, RDD, or PHD in OB and bMSC of OVX rats. No significant changes in either OPG or RANKL expression were observed in OB and bMSC of OVX rats treated with CSD.

**Conclusions:**

Our study showed that *RDD* and *PHD* increased bone formation by stimulating overexpression of OPG and downregulation of RANKL in OB and bMSC. This suggests that RDD and PHD may be used as alternative therapeutic agents for postmenopausal osteoporosis.

## Background

Osteoporosis, the most serious bone remodeling disease, is defined by low bone mass and a high risk of fractures. It mainly affects postmenopausal women and elderly men. Osteoporosis is caused by abnormal bone remodeling (i.e., access of resorption and less formation), resulting in an increased risk of hip and vertebral fracture [1]. Bone remodeling in healthy bone is an essential process for maintaining bone quality and repairing damaged bone that can adapt appropriately to mechanical stimuli. Osteoporosis is a condition in which loss of bone mass leads to fragility fractures. Postmenopausal osteoporosis caused by estrogen deficiency is characterized by a high-turnover state in bone remodeling; that is, bone resorption and formation were all maintained at a high level, but the bone resorption rate was higher than the bone formation rate in postmenopausal women [2].

The development of bone fragility in postmenopausal women results from changes in bone remodeling that leads to alteration of trabecular bone volume and architecture [3]. In rats, ovariectomy (OVX)-induced bone loss can be prevented by treatment with estradiol. Because of similarities in skeletal responses to estrogen deficiency between rats and humans, the mature OVX rat is considered a good animal model for studying early postmenopausal bone loss [4].

Hormone replacement therapy (HRT) is an established regime for prevention of postmenopausal bone loss [5], but recent evidence indicates that its long-term use is accompanied by side effects, such as the increased risk of breast, ovarian, and endometrial cancer [6]. Thus, alternative therapeutic strategies with proven efficacy and safety should be developed for the prevention and treatment of osteoporosis.

Chinese herbal medicine has been widely used in clinical practice to treat bone disease for thousands of years and will undoubtedly continue to be used as a cost-effective alternative medicine in China. Three Chinese herbs, *Radix Dipsaci* (RD), *Pyrola Herb* (PH), and *Cynomorium songaricum* (CS) have been used to reinforce the kidney-yang and strengthen bone. Although the therapeutic effects of these herbs on osteoporosis induced by estrogen deficiency are yet unknown, recent study suggests that *RD* crude extract could increase bone mass and alter bone histomorphology in rats [7]. The objective of this study was to evaluate the effects of *Radix Dipsaci* decoction (RDD), *Pyrola Herb* decoction (PHD), and *Cynomorium songaricum* decoction (CSD) on postmenopausal osteoporosis.

## Methods

### Identification and preparation of herb decoctions

*Radix Dipsaci* (RD) is the dried roots of *Dipsacus asperoides C. Y. Cheng et T. M. Ai* produced in China, and was collected by the Zhonghong Herbal Drug Co. Ltd. (Sichuan, China) in March 2007, and identified and authenticated by an expert herbalist at the Institute of Chinese Materia Medica, China Academy of Chinese Medical Sciences (CACMS).

*Pyrola Herb* (PH) is the dried aerial parts of *Pyrola calliantha H. Andr* produced in China, and was collected by the Huanghe Herbal Drug Co. Ltd. (Gansu, China) in March 2007, and identified and authenticated by an expert herbalist at the Institute of Chinese Materia Medica, CACMS.

*Cynomorium songaricum* (CS) is the dried fleshy stem of *Cynomorium songaricum Rupr* produced in China, and was collected by the Taizhou Herbal Drug Co. Ltd. (Zhejiang, China) in March 2007, and identified and authenticated by an expert herbalist at the Institute of Chinese Materia Medica, CACMS.

After collection, all the dried herbs were stored in a dry and sealed container at 4°C to protect them from moisture and moths.

Decoctions of RD, PH, and CS were extracted from the herbs by boiling 300 grams of the dried RD, PH, and CS in 6 liters of water at 100°C for 2 h. Each decoction was then concentrated to a final concentration of 1 crude drug gram per milliliter.

### Animals and experimental procedures

Sixty-nine virgin Wistar rats (3 months old) in a body weight range of 250 ± 20.0 grams were obtained from The Experimental Animal Center of the Academy of Military Medical Sciences (Beijing, China) and housed in cages at the experimental animal room at the Institute of Basic Theory of Traditional Chinese Medicine in CACMS. These rats were maintained at 22°C with a 12 h light/dark cycle and fed with a standard rodent chow diet (produced by The Animal Center of The Fourth Military Medical University, Xi’an, China) containing 0.9% calcium and 0.7% phosphate, with distilled water available *ad libitum*.

The acclimatized rats either underwent sham surgery (a faked operative intervention that omits the step thought to be therapeutically necessary) (sham, n = 12) or bilaterally ovariectomized (OVX, n = 57) using the dorsal approach [8]. Briefly, a surgical procedure on each of the anesthetized rats was performed with a single longitudinal skin incision on the dorsal midline at the level of the kidneys. Both ovaries were ligated and removed. Rats in the sham group underwent sham surgery, during which the ovaries were exposed and remained intact. The OVX rats were randomly divided into five groups: OVX control group (OVX, n = 12), positive control group treated with diethylstilbestrol (E2, n = 12), and three groups treated with RDD (n = 11), PHD (n = 11), and CSD (n = 11), respectively. Non-surgical rats (n = 12) were used as normal control (NC). The rats in the E2 group received diethylstilbestrol at a dose of 0.008 mg/ml (dissolved in distilled water) orally. The rats in the RDD, PHD, and CSD treatment groups were fed intragastrically with *RDD* at 5.6 ml per kg body weight a day, *PHD* at 5.6 ml per kg body weight a day, and *CSD* at 5.6 ml per kg body weight a day, respectively. This dose, about 40-fold lower than LD_50_ of these herbs (222.84 g/kg body weight), was optimized in our pre-experimental studies (data not shown). Rats in the NC, sham, and OVX control groups were administrated with an equal volume of distilled water to that in the decoction treatment groups. All the treatments were started four weeks after the surgery and continued for 12 weeks. The body weight of each rat was monitored weekly to assess the effect of the treatments.

On the fifteenth and third days before sacrifice, all the rats received tetracycline at 30 mg/kg by intraperitoneal injection. After sacrifice, the success of ovariectomy was confirmed at necropsy by failure to detect ovarian tissue and by observation of marked atrophy of the uterine horns.

All animals were maintained according to the *Guide for Care and Use of Laboratory Animals*, with the approval of the Institutional Ethics Committee of the CACMS.

### Bone histomorphometry

After sacrifice, the tibiae were dissected for histomorphometrical analysis. Proximal right tibiae were fixed in 4% paraformaldehyde for 24 h, then dehydrated in an ethanol gradient of 80%, 90%, and 100% for two days at each step. Dehydrated samples were defatted in xylene for two days before embedding in plastic polymer solution I (100 ml Methyl Methacrylate Monomer, 35 ml Butyl Methacrylate, 5 ml Methyl Benzoate, and 1.2 ml Polyglycol 1.2 ml), solution II (solution I, 0.4 gram Drying Benzoyl Peroxide), and solution III (solution II, 0.8 gram Drying Benzoyl Peroxide) for three days at each step. Each undecalcified tissue was sliced into two longitudinal sections at 5 μm thickness using microtome. One section was stained with toluidine blue, and the other was used for fluorescence morphology observation. Proximal left tibiae were fixed in 4% paraformaldehyde for 24 h, then decalcified in a decalcifying solution of 10% EDTA (pH7.3) at 4°C for three weeks. After that, the decalcified samples were dehydrated in 15% sucrose solution for 10 h. Each sample was sliced into sections of 5 μm thickness each by freezing microtome. Frozen sections were fixed in acetone and ready for use for analysis of immunohistochemistry and *in situ* hybridization.

Undecalcified tibial slides were used for analysis of bone remodeling activity. All measurements were performed using the Qwin Image analysis system (Leica Corporation, Bensheim, Germany). Six bone histomorphometric parameters were analyzed, including percentage of trabecular bone volume (TBV%), percentage of trabecular resorption surface (TRS%), percentage of trabecular formation surface (TFS%), mineralization rate of trabecula (MAR), mineralization rate of bone cortex (mAR), and osteoid mean width (OSW)

### Methylene blue staining

The undecalcified tibial sections were coated in Methyl Methacrylate Monomer solution for 2 h, then dehydrated in xylene solution for 1 h twice to remove resin. The samples were then rehydrated in an order of ethanol gradients, 100%, 90%, 80%, and 0% (in water). The rehydrated samples were stained with 1% toluidine blue, then rinsed with water. After that, the stained slides were dehydrated in an ethanol gradient of 80%, 90%, and 100%. Dehydrated slides were defatted in xylene, then sealed for microscopic analysis.

### Immunohistochemical analysis

Frozen sections were mounted onto glass slides and used for immunohistochemical analysis. Protein expression level of RANKL and OPG in tissue sections was estimated using rat antibodies of either anti-RANKL (1:1000) or anti-OPG (1:1000) (Santa Cruz Biotechnology, CA, USA). The sections were rinsed with TBS, then immersed in 0.3% hydrogen peroxide for 5 min. The slides were then incubated with specific antibodies for 1 h at 37°C and rinsed with TBS three times for 3 min. Sections were then incubated with the appropriate unbiotinylated secondary antibody (Zhongshan Goldenbridge Biotechnology Co. Ltd, Guangzhou, China) for 30 min at 37°C. Slides were then incubated with a solution containing DAB (1, 4-dideoxy-1,4-imino-d-arabinitol-diaminobenzidine; Sigma, USA) for 3 min and rinsed with running water, then counterstained with Harris hematoxylin and sealed for microscopic examination. Slides incubated with nonimmune goat serum, instead of the primary antibody, served as negative control.

Image analysis was performed using the Qwin Image analysis system (Leica Corp, Bensheim, Germany). Five images from each tibial section of a rat were randomly captured with 400× magnification. The relative level of the osteoblasts (OB) and bone marrow stromal cells (bMSC) in trabecular area was calculated as a percentage based on the positive stained area of either OB or bMSC over the trabecular area in the selected images.

### *In situ* hybridization

After a brief warm-up at room temperature, frozen sections were immersed in solution of 30% hydrogen dioxide and methanol for 30 min, then incubated with pepsin diluted by 3% citric acid at 37°C. After that, the sections were postfixed in 1% paraformaldehyde for 10 min. Sections were then incubated with the DIG-labeled antisense cRNA probes at 38°C-42°C overnight in a humidified chamber. After multiple washes in 4 × SSC at room temperature, slides were incubated in a blocking reagent for 30 min at 37°C, then with a biotinylated anti-digoxin antibody for 60 min, SABC for 20 min, and the biotinylated peroxydase for 20 min, at 37°C, followed by staining with DAB (Sigma-Aldrich Corp.). Finally, sections were covered with glycerol-gelatin and coverslips, and were ready for microscopic examination. The primer sequences used are listed below:

OPG:

Forward: 5'-TGGACAACCCAGGAAACCTTTCCTCCAAAA-3'

Reverse: 5'-TTTGCCTGGGACCAAAGTGAATGCAGAGAG-3'

Probe: 5'-AGAAATGATAGGGAATCAGGTTCAATCAGT-3'

RANKL:

Forward: 5'-GCCAGCCGAGACTACGGCAAGTACCTGCGC-3'

Reverse: 5'-GGCCAGGTGGTCTGCAGCATCGCTCTGTTC-3'

Probe: 5'-TTTATAGAATCCTGAGACTCCATGAAAACG-3'

### Statistical analysis

All values were expressed as mean ± standard deviations. All analyses were carried out using SPSS 12.0 (SPSS Inc., Chicago, IL, USA). The difference between the groups regarding the evaluated parameters was tested using the ANOVA test followed by the Tukey test. The data of all groups passed the Kolmogorov-Smirnov test of normality. All groups were carried out in triplicate and significance was accepted when P ≤ 0.05.

## Results

### Effects of RDD, PHD, and CSD on bone formation

**Figure 1:**
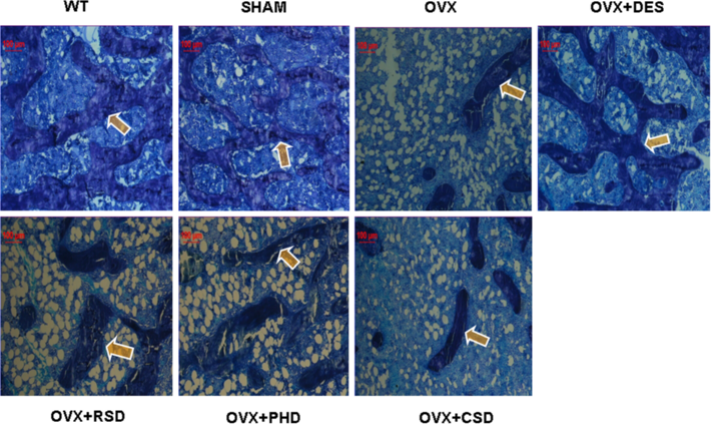
**Effects of RDD, PHD, and CSD on bone formation.** Trabecular bone formation was visualized by using methylene blue staining. See the details in the Methods section of the text.

To investigate the effects of the herbal medicines on overall bone formation, we analyzed trabecular bone formation in the undecalcified tibial sections from tibiae of OVX rats treated with RDD, PHD, and CSD using methylene blue staining (Figure 1). Trabecular bone formation was estimated by numbers of stained trabeculae in the undecalcified section. Figure 1 shows that trabecular bone formation was reduced significantly in the ovariectomized rats compared to rats in normal control and sham groups, while this effect was remarkably reversed by E2 treatment. Numbers of trabeculae in the stained tibial section were significantly increased in OVX rats treated with RSD and PHD, but not with CSD (Figure 1), compared to that in OVX control.

### Effects of RDD, PHD, and CSD on bone histomorphometry

Bone remodeling is a lifelong process in which two counter-balanced processes (bone resorption and bone formation) are involved. Bone remodeling activity can be monitored by measuring histomorphometric indices and bone remodeling markers. To further estimate the effects of RDD, PHD, and CSD on bone remodeling activity, we analyzed six bone histomorphometric parameters, including TBV%, TRS%, TFS%, MAR, mAR, and OSW in OVX rats treated with the Chinese herbs and E2 using histomorphometry (Table 1). TBV% was significantly reduced (*p* < 0.01) in rats with estrogen deficiency induced by ovariectomy. Yet, TRS%, TFS%, MAR, mAR (*p* < 0.01) and OSW (*p* < 0.05) were increased significantly in these OVX rats.

**Table 1:** Effects of RDD, PHD, or CSD on bone histomorphometry in rats treated for 12 weeks

**Group**	**n**	**TBV%**	**TRS%**	**TFS%**	**MAR(μm/d)**	**mAR(μm/d)**	**OSW(μm)**
Sham	12	27.18 ± 8.78	3.40 ± 1.54	7.40 ± 2.41	1.38 ± 0.16	2.28 ± 0.47	6.20 ± 1.29
OVX	12	8.945 ± 3.04^a^	9.31 ± 2.22^a^	14.54 ± 3.31^a^	1.86 ± 0.23^a^	3.03 ± 0.60^a^	7.77 ± 1.64^b^
E2	12	23.61 ± 4.71^c^	3.28 ± 1.31^c^	7.72 ± 2.66^c^	1.32 ± 0.22^c^	2.15 ± 0.70^c^	6.37 ± 1.42^d^
RDD	11	12.43 ± 2.96^d^	5.71 ± 1.72^c^	11.26 ± 2.97^d^	1.59 ± 0.25^d^	2.77 ± 0.42	7.35 ± 1.14
PHD	11	18.58 ± 5.53^c^	4.63 ± 1.42^c^	12.44 ± 3.26	1.95 ± 0.36	2.84 ± 0.71	7.86 ± 1.79
CSD	11	11.95 ± 3.87	8.60 ± 2.23	15.20 ± 4.11	1.68 ± 0.35	2.81 ± 0.49	7.80 ± 1.46

Values are mean ± S.D.

^a^*p* < 0.01 different from sham control group.

^b^*p* < 0.05 different from sham control group.

^c^*p* < 0.01 different from the OVX control group (one-way ANOVA).

^d^*p* < 0.05 different from the OVX control group (one-way ANOVA).

E2 treatment significantly reversed the effects of ovariectomy on histomorphometric indices by decreasing TBV% and increasing TRS%, TFS%, MAR, mAR, and OSW in OVX rats. Either RDD or PHD treatment showed a similar effect to E2 treatment on bone histomorphometric indices. However, CSD treatment had no significant effect on the six indices (*p* > 0.05) (Table 1).

### Effects of RDD, PHD, and CSD on protein expression of OPG and RANKL

**Figure 2:**
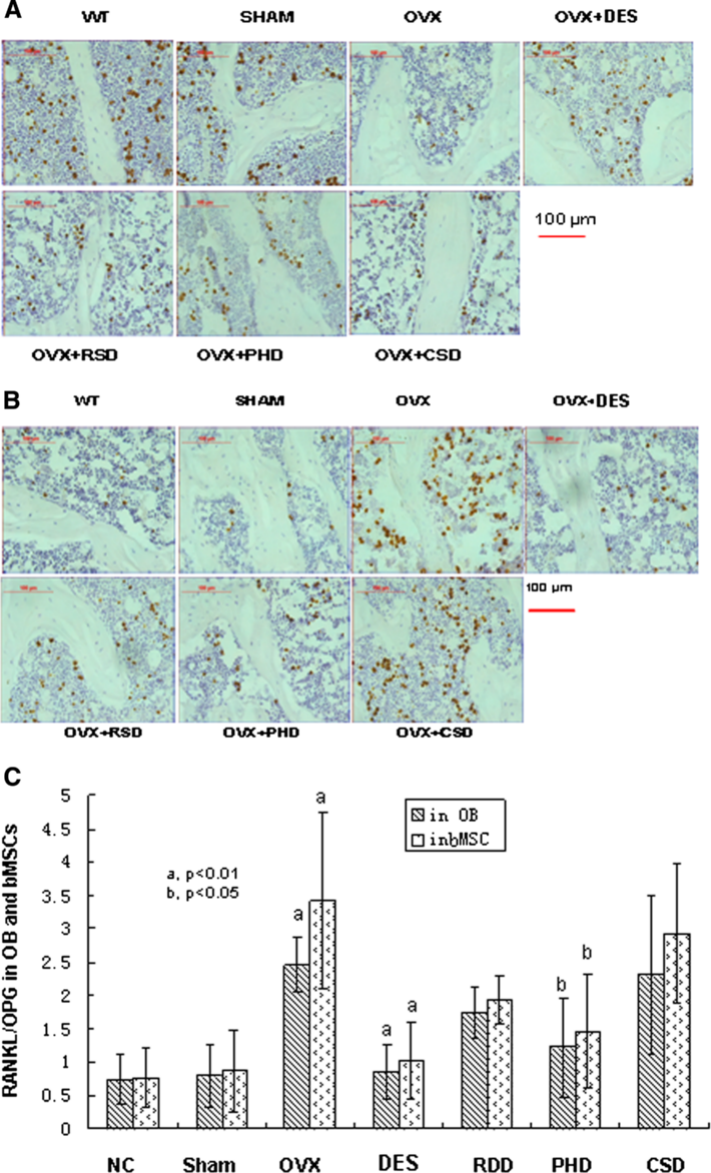
**Effects of RDD, PHD, and CSD on protein expression of OPG and RANKL. A**) Immunohistochemical stain of OPG; **B**) Immunohistochemical stain of RANKL; **C**) Ratio of RANKL/OPG protein expression, RANKL and OPG protein expression data were obtained from panels **A** and **B** of this figure, see details in the Methods section of the text, (a, p < 0.01, b, p < 0.05). All the experiments were repeated three times.

To monitor bone remodeling activity in OVX rats treated with the herbs, we analyzed biomarker expression of bone remodeling using immunohistochemistry (Figures 2A, B). OPG expression was decreased significantly in either OB or bMSC of tibia from OVX rats compared to the sham group (*p* < 0.01, Figure 2A). Either E2 or PHD treatment caused overexpression of OPG in OB and bMSC of tibia from OVX rats compared to that from the OVX control group (*p* < 0.05, Figure 2A); however, treatment with either RDD or CSD had no significant effect on OPG expression (*p* > 0.05).

We also analyzed RANKL expression in OB or bMSC of tibia from OVX rats treated with RDD, PHD, CSD, or E2 for 12 weeks of treatment using immunohistochemistry. Ovariectomy caused a significant increase in RANKL expression in OB or bMSC of tibia compared to that from the sham group (*p* < 0.01, Figure 2B). Expression of RANKL in OB or bMSC of tibia from OVX rats decreased significantly upon treatment with E2, RDD, or PHD (*p* < 0.01, *p* < 0.05, and *p* < 0.05, respectively, Figure 2B), but not with CSD (*p* > 0.05).

The ratio of RANKL/OPG in OB and bMSC of tibia from OVX rats was increased significantly compared to that in the normal control or sham groups (*p* < 0.01, Figure 2C). Compared to OVX alone, the ratio of RANKL/OPG decreased remarkably in OB and bMSC of tibia from OVX rats treated with either E2 (*p* < 0.01) or PHD treatment (*p* < 0.05); however, no significant changes in the ratio of RANKL/OPG were observed in OB and bMSC of tibia from OVX rats treated either with RDD or CSD (Figure 2C).

### Effects of RDD, PHD, and CSD on mRNA expression of RANKL and OPG

**Figure 3:**
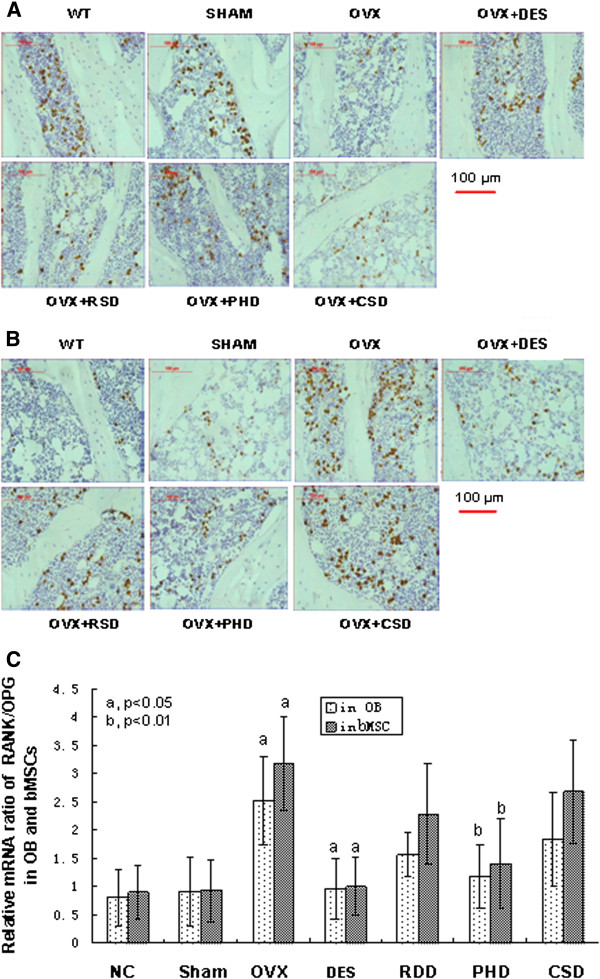
**Effects of RDD, PHD, and CSD on expression of OPG and RANKL mRNA. A**) *In situ* hybridization of OPG in OB and MSC; **B**) *In situ* hybridization of RANKL in OB and bMSC; **C**) Ratio of RANKL/OPG mRNA expression, RANKL and OPG mRNA expression data were obtained from panels **A** and **C** of this figure, see details in the Methods section of the text, (a, p < 0.01, b, p < 0.05). All the experiments were repeated three times.

To determine whether RDD, PHD, and CSD caused mRNA changes in RANKL and OPG in OB and bMSC of tibia from OVX rats, we examined mRNA levels of RANKL and OPG using *in situ* hybridization. OVX resulted in a significant decrease in expression of OPG mRNA in either OB or bMSC of tibia from OVX rats compared to the sham group (Figure 3A). The level of OPG mRNA in both OB and bMSC of tibia from OVX rats was induced significantly by treatment with either E2 or PHD, but no significant changes of OPG mRNA were observed with treatment with either RDD or CSD compared to that in the OVX control group (Figure 3A).

Expression of RANKL mRNA in OB or bMSC of tibia from the OVX rats was increased significantly compared to either the NC or sham groups (Figure 3B). Expression of RANKL mRNA was decreased remarkably in OB and bMSC of tibia from OVX rats treated with either E2 or PHD (Figure 3B), but no significant difference in RANKL mRNA was observed in OB and bMSC of tibia from OVX rats treated with either RDD or CSD.

The ratio of RANKL/OPG mRNA in OB and bMSC of tibia from OVX rats was increased significantly compared to that in normal control or sham groups (*p* < 0.01, Figure 3C). Compared to OVX alone, the ratio of RANKL/OPG mRNA decreased remarkably in OB and bMSC of tibia from OVX rats treated with either E2 (*p* < 0.01) or PHD (*p* < 0.05); however, no significant changes in the ratio of RANKL/OPG were observed in OB and bMSC of tibia from OVX rats treated with either RDD or CSD (Figure 3C).

## Discussion

*RD*, *PH*, *CS,* and other kidney-tonifying traditional Chinese medicines (TCM) have been widely used for thousands of years to treat bone diseases in China. The theory of Chinese medicine is to restore balance at all effective levels. Although these herbal medicines are considered a cost-effective alternative by Chinese people, lack of knowledge about their mechanisms limited their application as an alternative therapeutic regime to treat osteoporosis in most Western countries.

Previous studies showed that ovariectomy induced bone loss, resulting in an increased risk of osteoporosis. Our results from this study confirm this notion, indicating that the mature OVX rat is a good animal model of osteoporosis for evaluating the efficacy of herbal medicines as therapies for early postmenopausal osteoporosis.

Recently, several studies of herbal medicines have been performed to evaluate their antiosteoporotic effects, suggesting that herbal medicines may be developed as useful alternative medicines for treatment of osteoporosis [9,10]. Shirwaikar et al. used ethanol extract of *Cissus quadrangularis* (CQ) to treat an ovariectomized rat model of osteoporosis, and found that CQ ethanol extract of the plant had a definite anti-osteoporotic effect [9]. Chae’s group studied the anti-osteoporotic effect of the herbal medicine *Salvia miltiorrhiza* (SM), showing that SM ameliorated the decrease in BMD and trabecular bone mass of OVX-induced osteoporosis rats and decreased the serum level of osteoclast markers and oxidative stress markers. Based on the property of anti-oxidative stress, SM may be developed as a promising natural therapy for osteoporosis [10]. So far, there has been no report about the anti-osteoporotic effects of *Radix Dipsaci* (RDD), *Pyrola Herb* (PHD) and *Cynomorium songaricum* decoctions (CSD), as used in this study, in which the effects of these medicines on an ovariectomized rat model of osteoporosis were evaluated.

Although E2 is no longer used in clinical practice due to its side effects, use of E2 as control in this study is to help us understand the mechanism of action of the herb medicines in OVX rats. As expected, OVX rats treated with E2 for 12 weeks ameliorated the loss of bone mass, which was reflected by the increased TBV% and the decreased levels of TRS%, TFS%, MAR, OSW, and mAR. The effect of either RDD or PHD on TBV% and TRS% was similar to that of E2; however, the effect of either RDD or PHD on mAR and OSW of tibia from OVX rats was not significant, suggesting that either RDD or PHD may have a limited effect on bone cortex mineralization and osteoid formation. In addition, OVX rats treated with PHD did not show significant decreases in TFS% and MAR, suggesting that a possible anti-osteoporotic mechanism of PHD may be through inhibition of bone resorption.

It is well known that the RANKL/RANK/OPG signaling pathway plays a key role in the regulation of bone remodeling [11]. RANK is a receptor located on surface osteoclasts (precursor and mature). Ligands of RANK are OPG and RANKL, synthesized and secreted primarily by osteoblasts and bone marrow stromal cells [12]. Activation of RANK by RANKL initiates a RANK/RANKL/OPG signaling cascade, resulting in osteoclast differentiation and increased bone resorption. OPG, which acts as a decoy receptor for RANKL, blocks this interaction. The importance of this pathway in bone metabolism is demonstrated by the facts that pharmacologic blockage of RANKL is an effective treatment for osteoporosis [13], an inherited deficiency of RANK or RANKL causes osteopetrosis, and loss-of-function osteoprotegerin mutations cause juvenile Paget’s disease [14,15]. Different from previous studies [9,10], this study’s purpose was to answer how herbal medicines regulate the major bone remodeling pathway. We found that OVX could downregulate protein and mRNA expression of OPG and upregulate protein and mRNA expression of RANKL in OB or bMSC compared with the sham group, while E2 or PHD treatment could reverse this condition significantly. RDD could only significantly downregulate protein and mRNA expression of RANKL and had no significant effect on protein and mRNA expression of OPG. The results indicated that one of mechanisms at work in the inhibition of osteopenia by E2, PHD, and RDD lay in their modulation of the effects on OPG and RANKL.

## Conclusion

By comparison, this study demonstrated for the first time the potential protective effects of RDD and PHD on OVX-induced osteoporosis in rats. RDD and PHD could prevent the OVX-induced loss of bone mass; their preventive effects depended on modulating protein and mRNA expression of OPG and (or) RANKL in OB and bMSC. Our study provides evidence that *Radix Dipsaci* and *Pyrola Herb* extract have the potential to be used for the treatment of postmenopausal osteoporosis.

## Abbreviations

CS: (*Cynomorium songaricum*); CSD: (*Cynomorium songaricum* decoction); bMSC: (Bone marrow stromal cells); MAR: (Mineralization rate of trabeculae); mAR: (Mineralization rate of bone cortex); OB: (Osteoblasts); OPG: (Osteoprotegerin); OSW: (Osteoid mean width); OVX: (Ovariectomy); PH: (*Pyrola Herb*); PHD: (*Pyrola Herb* decoction); RD: (*Radix Dipsaci*); RDD: (*Radix Dipsaci* decoction); TBV: (Trabecular bone volume); TFS: (Trabecular formation surface); TRS: (Trabecular resorption surface).

## Competing interests

The authors declare that they have no competing interests.

## Authors' contributions

ML carried out rat experiments and drafted the manuscript. GGX and DJ were heavily involved in experimental design, and GGX was also mainly involved in scientific correction of the draft manuscript. PR, ZZ, JD, HZ, HL, YL, JP, HL, WW, and PZ were involved in sample collection and measurements of bone morphometry, as well as bone markers. All authors were involved in drafting the manuscript and revising it for critically important content. All authors have read and approved the final manuscript.

## Pre-publication history

The pre-publication history for this paper can be accessed here:

http://www.biomedcentral.com/1472-6882/12/67/prepub
